# Spin Glass State in Strained La_2/3_Ca_1/3_MnO_3_ Thin Films

**DOI:** 10.3390/nano12203646

**Published:** 2022-10-18

**Authors:** Irene Lucas, Noelia Marcano, Thomas Prokscha, César Magén, Rubén Corcuera, Luis Morellón, José M. De Teresa, M. Ricardo Ibarra, Pedro A. Algarabel

**Affiliations:** 1 Instituto de Nanociencia y Materiales de Aragón (INMA), CSIC-Universidad de Zaragoza, 50009 Zaragoza, Spain; 2 Departamento de Física de la Materia Condensada, Universidad de Zaragoza, 50009 Zaragoza, Spain; 3 Centro Universitario de la Defensa, Academia General Militar, 50090 Zaragoza, Spain; 4 Laboratory for Muon Spin Spectroscopy, Paul Scherrer Institut, CH-5232 Villigen PSI, Switzerland; 5 Laboratorio de Microscopías Avanzadas (LMA), Universidad de Zaragoza, 50018 Zaragoza, Spain

**Keywords:** strain engineering, manganites, epitaxial thin films, phase segregation, exchange bias, LE-μSR, magnetic relaxation, spin-glass-like state

## Abstract

Epitaxial strain modifies the physical properties of thin films deposited on single-crystal substrates. In a previous work, we demonstrated that in the case of La_2/3_Ca_1/3_MnO_3_ thin films the strain induced by the substrate can produce the segregation of a non-ferromagnetic layer (NFL) at the top surface of ferromagnetic epitaxial La_2/3_Ca_1/3_MnO_3_ for a critical value of the tetragonality τ, defined as *τ* = |*c* − *a*|*a*, of τ_C_ ≈ 0.024. Although preliminary analysis suggested its antiferromagnetic nature, to date a complete characterization of the magnetic state of such an NFL has not been performed. Here, we present a comprehensive magnetic characterization of the strain-induced segregated NFL. The field-cooled magnetic hysteresis loops exhibit an exchange bias mechanism below T ≈ 80 K, which is well below the Curie temperature of the ferromagnetic La_2/3_Ca_1/3_MnO_3_ layer. The exchange bias and coercive fields decay exponentially with temperature, which is commonly accepted to describe spin-glass (SG) behavior. The signatures of slow dynamics were confirmed by slow spin relaxation over a wide temperature regime. Low-energy muon spectroscopy experiments directly evidence the slowing down of the magnetic moments below ~100 K in the NFL. The experimental results indicate the SG nature of the NFL. This SG state can be understood within the context of the competing ferromagnetic and antiferromagnetic interactions of similar energies.

## 1. Introduction

Strain engineering has become one of the most popular routes to optimize the physical properties of thin film oxides grown on single-crystal substrates [1,2,3,4,5]. Among the complex oxides, manganites are good candidates for the tuning of the physical properties by the control of the electron occupancy of the Mn 3d orbitals via strain. This is due to the fact that the physics of these materials is governed by the geometry of the MnO_6_ octahedra in the perovskite structure. The strain-induced elongation, compression, or rotation of the MnO_6_ units lead to crystal field splitting of the x^2‒^ y^2^ and 3z^2^‒r^2^ levels, thus modifying their electron occupancy and leading to complex orbital reconstruction [5,6,7]. In this context, Marín et al. [8] showed the first direct observation of the strain-induced segregation of a non-ferromagnetic layer (NFL) at the top surface of ferromagnetic (FM) epitaxial La_2/3_Ca_1/3_MnO_3_ (LCMO) thin films as a function of the growth conditions, with the whole film being chemically and structurally homogeneous at room temperature. That was achieved by imaging the magnetization state of the films at nanometer scale below the Curie temperature (*T_C_*) of the FM LCMO layer using electron holography (EH). A comprehensive study using different single-crystal substrates, together with the fine-tuning of the growth conditions, revealed that the room-temperature tetragonality of LCMO, defined as τ = |c-a|/a, where *a* and *c* are the in-plane and out-of-plane lattice parameters of the LCMO film, respectively, determined the magnetic nature of the films. The coexistence of the NFL and FM phases at low temperature was found above a critical value of *τ_C_* ≈ 0.024.

Such a strain-induced NFL was characterized as antiferromagnetic (AFM) in nature based on the presence of exchange bias (EB) in the magnetic hysteresis loops at low temperatures (10 K) in the single LCMO film. The EB effect refers to a shift of the magnetic hysteresis loop along the field axis, which is often observed for heterostructures where FM and AFM materials are in contact. The basic mechanism of the exchange-bias effect is that the magnetization direction in an FM layer can be pinned by an adjacent AFM layer [9].

In the case of the LCMO films, the strain-induced crystal distortion was proposed as the physical mechanism explaining the segregation of the top AFM layer. In-plane compressive or tensile epitaxial strain in La_2/3_Sr_1/3_MnO_3_ (LSMO) favors the 3z^2^‒r^2^ (the C-type antiferromagnetic (AFM)) or the x^2^‒y^2^ (the A-type AFM) orbital ordering [6,10], respectively. As a consequence, the interface with the substrate or the surface of an FM (conductor) manganite could have a tendency toward AFM (insulator).

It should be highlighted that despite EB being commonly observed when an FM is in contact with an AFM due to the exchange coupling at the interface, further studies have revealed that this is a phenomenon of greater generality. Thus, Ali et al. reported EB in the Co/CuMn (FM/SG) bilayer metallic system, and it was suggested that the EB properties of the FM/SG system differ from those of the usual AFM/FM exchange-biased systems [11]. Cui et al. showed that EB in LSMO single films was due to the exchange coupling between FM LSMO and an unprecedented LSMO-based spin glass [12]. The EB was also reported to exist at the paramagnetic/ferromagnetic systems, such as paramagnetic LaNiO_3_-based heterostructures [13,14], and in epitaxial iridate-manganite heterostructures [15]. It is worth mentioning that in these cases, the physical mechanism for this unconventional EB effect is still under investigation. More recently, Maniv et al. [16] revealed the presence of a giant EB field in a disordered antiferromagnetic state hosted in the single-crystalline Fe_x_NbS_2_. In that case, the EB was due to coupling between the coexisting AFM and SG orders.

In this paper, we report a comprehensive magnetic characterization by means of magnetization measurements and low-energy muon spin spectroscopy of the NFL-FM phase segregation of a strained LCMO film to gain more insight into the origin of the EB effect observed at low temperatures and the magnetic nature of the NFL. Our systematic study of the magnetic properties indicates the spin-glass (SG) nature of the segregated NFL reported on this film. For this purpose, we studied a representative LCMO thin film deposited on SrTiO_3_ (STO).

## 2. Materials and Methods

Sixty-nanometer-thick LCMO film was grown onto (100)-oriented STO single-crystal substrate using pulsed laser deposition (PLD) at a laser repetition rate of 10 Hz, resulting in a tetragonality value of *τ* = 0.025 > *τ_C_*. The structural characterization was performed by X-ray diffraction (XRD), scanning transmission electron microscopy (STEM), and electron energy-loss spectroscopy (EELS). STEM-EELS was carried out in a probe-corrected Thermo Fisher Titan 60-300 microscope installed at the Laboratorio de Microscopías Avanzadas (LMA), at University of Zaragoza. Further details on the deposition conditions and structural characterization were reported elsewhere [8].

The magnetic properties were measured in a commercial (Quantum Design, CA, USA) superconducting quantum interference (SQUID) magnetometer (QD-MPMS). The applied magnetic field was always parallel to the film plane and along the (100) direction of the STO substrate. The field-cooled (FC) and zero-field-cooled (ZFC) magnetization measurements were made on heating from 5 to 300 K after the sample was cooled from 300 K down to the selected temperature with and without the magnetic field, respectively. The isothermal hysteresis loops were measured after FC from 300 K under a 0.1 kOe applied magnetic field. For the measurement of the relaxation of the thermal remnant magnetization, the sample was field cooled under a magnetic field of 0.1 kOe from the paramagnetic state to the selected temperature, and then, the time dependence of the magnetization was measured after the magnetic field was off. Electron holography experiments were performed at 100 K in an image-corrected Thermo Fisher Titan Cube 60–300 microscope installed at the Laboratorio de Microscopías Avanzadas (LMA), at University of Zaragoza. Additional details on holography experiments and data analysis can be found elsewhere [8].

LE-*μSR* experiments were performed using the low-energy muon spectrometer (Paul Scherrer Institute, PSI, Switzerland). The muon implantation depth in the film was determined by the Monte Carlo simulation program TRIM.SP [17]. The measurements were performed in the temperature range of 5–300 K.

## 3. Results and Discussion

**Figure 1:**
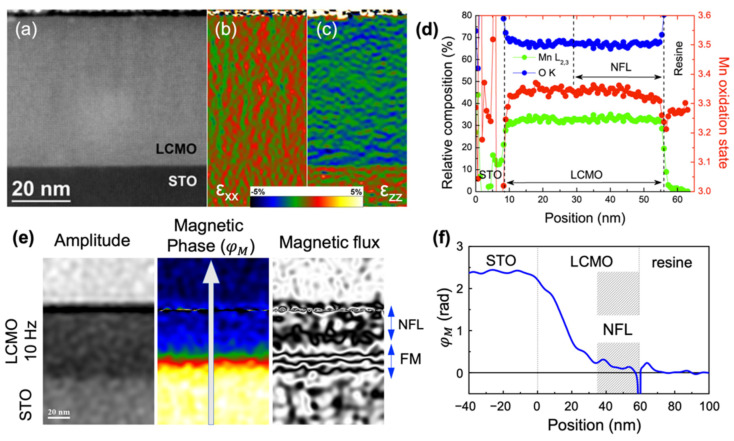
Structural, chemical, and magnetic characterization of the NFL of the LCMO thin film. (**a**) Cross-sectional HAADF-STEM image of the LCMO film grown on STO (100). (**b**,**c**) Relative deformation of the in-plane (**b**) and the out-of-plane (**c**) lattice parameter of the LCMO with respect to the substrate obtained by the geometrical phase analysis of the image shown in (**a**). (**d**) STEM-EELS compositional line profile and estimated Mn oxidation state across the LCMO film. (**e**) Amplitude of the electron wave, magnetic phase shift (φ_M_), and magnetic flux distribution of the LCMO film obtained by off-axis electron holography. (**f**) Line profile of φ_M_ extracted along the white arrow in (**e**). Adapted with permission from Ref. [8]. Copyright 2015 American Chemical Society.

Figure 1 summarizes the structural, chemical, and magnetic characterization of the NFL of the LCMO thin film. Local STEM characterization shows that the LCMO film is fully strained and structurally and chemically homogeneous. However, electron holography experiments performed at 100 K evidence the onset of an NFL, approximately 24 nm-thick, on the surface region of the LCMO, while the rest of the film remains FM.

**Figure 2:**
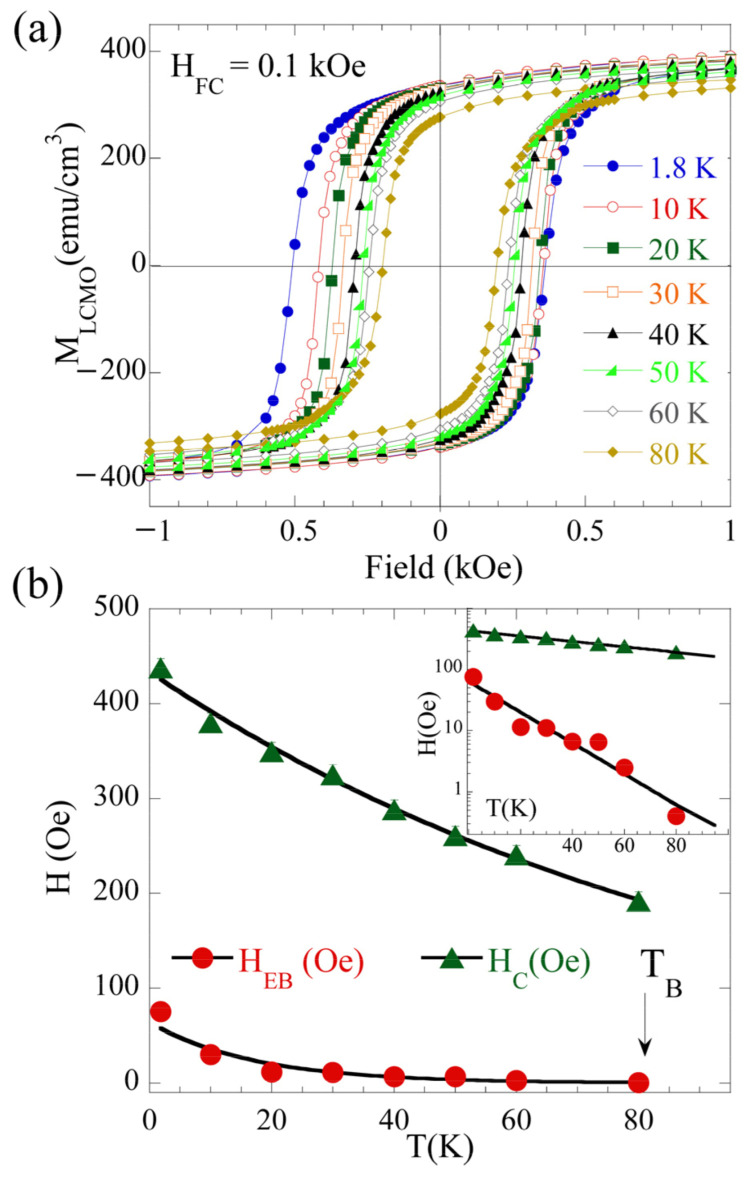
(**a**) Magnetic hysteresis loops for the LCMO film measured at different temperatures after being field cooled in 0.1 kOe from room temperature. For clarity, only the data between −1 and 1 kOe are shown, while the measurements were performed between −50 and 50 kOe. (**b**) Temperature dependence of H_EB_ and H_C_ for the LCMO film. The solid lines are fits to the exponential temperature dependencies for SG behavior described in the text. Error bars are either of the same magnitude or smaller than the point size. The inset shows both the temperature dependence of H_EB_ and H_C_ and the exponential fits in logarithmic scale (see text for details). The arrow marks the characteristic temperature where EB vanishes (T_B_).

To reveal the origin of the EB in the single LCMO film, the temperature dependence of the exchange bias parameters, namely the exchange-bias field (H_EB_) and the coercive field (H_C_), has been studied. The EB field is defined as $H_{EB}=\frac{\left|H^{+}+H^{-}\right|}{2}$ and the coercive field $H_{C}=\frac{\left|H^{+}-H^{-}\right|}{2}$, where *H^+^* and *H^−^* denote the right and left coercive fields in the magnetic hysteresis loop, respectively. For such measurements, the sample was cooled down from room temperature to the predetermined temperature with an applied field H_FC_ = 0.1 kOe. This process was repeated for every measuring temperature. The magnetic hysteresis loops recorded at several temperatures (T = 1.8, 10, 20, 30, 40, 50, 60, and 80 K) are depicted in Figure 2a and are representative of this study. The loops were performed by ranging the magnetic field between −50 and +50 kOe in the plane of the film. For the sake of clarity, only the data between −1 and 1 kOe are shown. Figure 2b shows the temperature-dependent trend of the extracted H_EB_ and H_C_ at low temperatures. Both exhibit an exponential decrease as a function of temperature.

Such an exponential thermal decay is considered as a fingerprint of the fact that the origin of the H_EB_ and H_C_ is due to the existence of spin frustration; this was previously reported in other systems, including oxides and metals, such as LSMO/LaNiO_3_ bilayers [18]; LSMO/SMO bilayers [1,19]; LSMO/SrIrO_3_(SIO) bilayers [15]; LSMO/SMO/LSMO trilayers [20]; La_1-x_Ca_x_MnO_3_ FM (x = 0.33)/AFM (0.67) multilayers [21]; FM/SG Ni/Ni_76_Mn_24_ bilayers [22]; amorphous/crystalline NiFe_2_O_4_ ferrite [23]; NiMn/CoFeB bilayers [24]; and Co/CuMn bilayers [11], where the interfacial spin-glass state plays an important role in the EB effect. Thus, the temperature dependence of H_EB_ and H_C_ can be fitted using the phenomenological formulas $H_{EB}=H_{EB}^{0}e^{-T/T_{1}}$ and $H_{C}=H_{C}^{0}e^{-T/T_{2}}$, where $H_{EB}^{0}$ and $H_{C}^{0}$ are the extrapolation of H_EB_ and H_C_ at zero temperature; *T_1_* and *T_2_* are constants. The solid lines in the Figure 2b show those fits with the fitting parameters *H_EB_*^0^ = 64(6) Oe, *T*_1_ = 17(1) K, *H_C_^0^* = 433(6) Oe, and *T_2_* = 99(3) K. Exchange-biasing H_EB_ vanishes at a characteristic temperature *T_B_* of about 80 K. As will be shown below, this temperature is much lower than the magnetic ordering temperature of the FM LCMO (*T_C_*).

**Figure 3:**
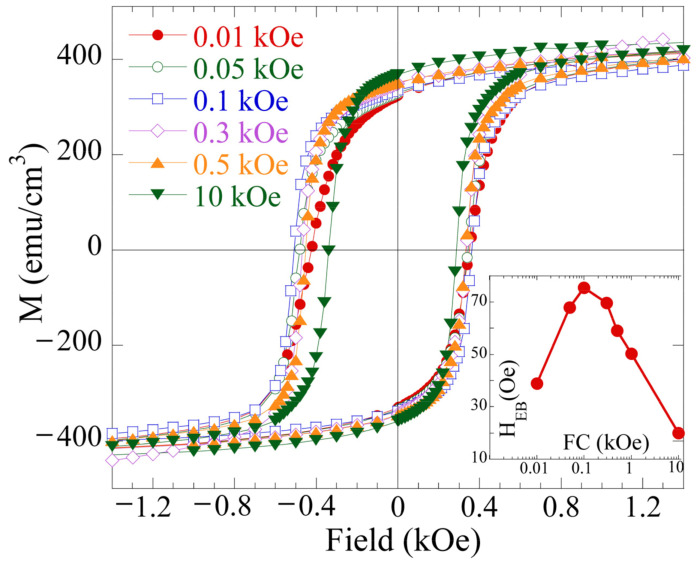
Magnetic hysteresis loops measured at 1.8 K after field cooling from room temperature in different magnetic fields of 0.01, 0.05, 0.1, 0.3, 0.5, 1, and 10 kOe. H_EB_ is shown as a function of the cooling field in the inset. The solid line is a guide for the eye.

To investigate the effect of the cooling field H_FC_ on the exchange-bias parameters H_EB_ and H_C_, the hysteresis loops at 1.8 K were measured with various cooling fields (H_FC_ = 0.01, 0.05, 0.1, 0.3, 0.5, 1, and 10 kOe) (Figure 3). H_EB_ as a function of H_FC_ is summarized in the inset of Figure 3. H_EB_ first shows a rapid increase with increasing H_FC_ with a maximum H_EB_ of 80 Oe at H_FC_ = 0.1 kOe and then decreases down to 20 Oe after cooling in a field of 10 kOe. A similar trend in H_EB_ on H_FC_ was also reported in other systems exhibiting interfacial spin frustration, such as LaSrMnO_4_/LSMO [12], LSMO/SIO bilayers [15], and LaMnO_3_/SrMnO_3_ [25]. According to this evidence, the EB effect of the NFL-FM LCMO can be understood in terms of the FM/SG interface. In zero-field cooling, the moments are randomly distributed without any net polarization. However, when cooling in fields from room temperature, the moments in the SG layer will gradually align with the cooling field, inducing a net polarization on the SG state. The SG state will freeze below a characteristic blocking temperature *T_B_*. With temperatures further decreasing below *T_B_*, the frozen net polarization of the SG state leads to a unidirectional anisotropy, necessary for the occurrence of the EB. The higher the cooling field is, the larger is the induced net polarization of the SG state. This scenario could explain the rapid increase in H_EB_ with H_FC_ shown in Figure 3 for the H_FC_ values lower than 0.1 kOe. The decrease in H_EB_ under a cooling field higher than 0.1 kOe could be explained by the existence of FM and AFM states of similar energies, as is demonstrated by the DFT+U calculations of the magnetic ordering in strained LCMO [8]. The presence of a relatively high cooling field could favor the FM phase to the detriment of the AFM phase, thus weakening the SG phase, originated by the competition between the FM and AFM interactions, and therefore reducing the H_EB_ field.

**Figure 4:**
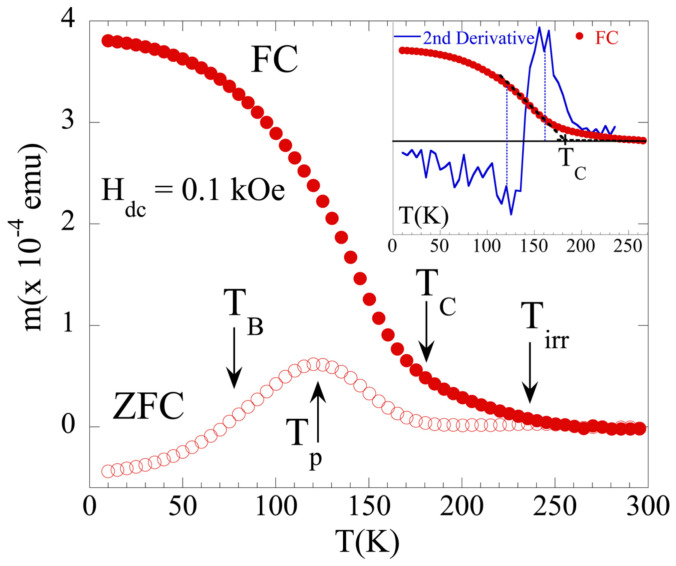
Temperature dependence of ZFC (open symbols) and FC (solid symbols) magnetization in an applied field of 0.1 kOe for the LCMO film. The characteristic temperature where EB vanishes (*T_B_*) is also displayed. The inset shows schematic of two-tangent method for determination of the *T_C_* [26] (see text for details). The vertical lines serve as a guide to estimate the temperature regime of the first straight line.

Figure 4 shows the field-cooling (FC) and zero-field-cooling (ZFC) magnetization versus temperature *(M-T)* curves measured with an in-plane field of 0.1 kOe. For the FC and ZFC magnetization measurements, the sample was cooled down from the paramagnetic state with and without the magnetic field, respectively. The cooling field, H_FC_, was 0.1 kOe with the same direction as the measuring field. Both the ZFC and the FC *M-T* curves were measured while warming the sample. The ferromagnetic transition temperature *T_C_* for the FM LCMO film was estimated using the Grommé’s method [26], from the intercept of two straight lines fitted to the magnetization curve on either side of the *T_C_* inflection point. A schematic of the two-tangent method for the determination of *T_C_* is displayed in the inset of Figure 4. This was performed by fitting the first straight line to the magnetization curve from halfway between the minimum and the zero point of the second derivative of the magnetization curve to halfway between the zero point and the maximum in the second derivative of the magnetization curve. The second straight line was fitted to the magnetization curve between 280 and 300 K. The intersection of these lines occurs at *T_C_* = 183(5) K (see inset in Figure 4), in agreement with previous studies [27]. There are three major highlights in the ZFC-FC *M-T* data: (i) a pronounced bifurcation between the ZFC and FC branches below a characteristic irreversibility temperature *T_irr_* = 240(1) K, well above the *T_C_* of the FM LCMO layer (*T_irr_*~1.4*T_C_*); (ii) a maximum in the ZFC *M-T* curve at *T_p_*~120 K, well below *T_C_*; and (iii) a strong enhancement of the magnetic irreversibility/bifurcation in the ZFC and FC curves just below *T_P_*. Here, ZFC smoothly decreases, whereas the FC branch smoothly increases.

The origin of *T_irr_* is attributed to the presence of magnetic polarons as reported in the LCMO bulk above the ferromagnetic ordering temperature, *T_C_* [28]. According to De Teresa et al., this temperature corresponds to the onset of short-range ferromagnetic correlations (clusters). In the present case, *T_irr_* would be indicative of the appearance of ferromagnetic clusters in the FM LCMO layer [29,30].

The enhancement of the difference between the ZFC and the FC *M(T)* curves, resulting in the pronounced maximum in the ZFC curve at *T_P_* = 120 K, is a typical feature observed in magnetically disordered systems, including spin glasses [31] and others, such as superparamagnets and cluster glasses. The ZFC spin glass will be gradually frozen into a random distribution of magnetic moments, leading to a decrease in the magnetization with the temperature, which gives rise to the magnetization peak observed at *T_p_*. For the FC spin glass, however, it will be partially polarized by the cooling field and a net magnetization will be induced, giving rise to the bifurcation between the ZFC and FC *M(T)* curves. Therefore, the observed bifurcation, together with the experimental observations presented above, points towards the emergence of spin-glass-like behavior in this film. In the present case such an irreversibility occurs above *T_B_* (~80 K), the characteristic temperature below which EB is detected in the hysteresis loops. Furthermore, the ZFC *M-T* signal from the LCMO film strongly decreases below *T_B_*, whereas the FC *M-T* branch smoothly increases. The low negative magnetization in the ZFC data observed below 50 K may be due to the diamagnetic contribution from the substrate.

**Figure 5:**
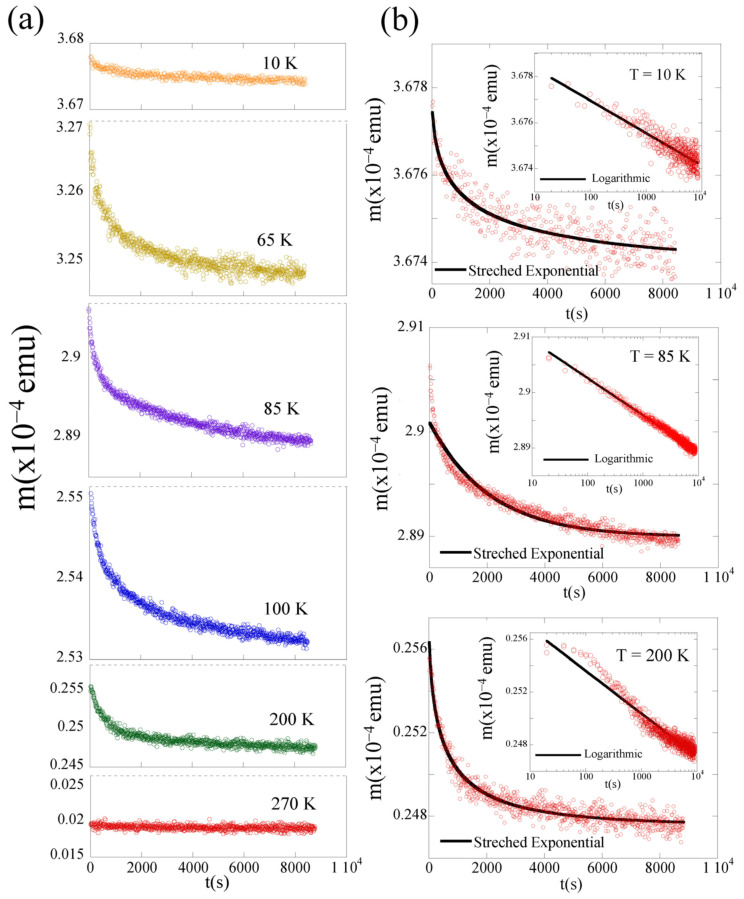
(**a**) Time dependence of TRM after field cooling under a magnetic field of 0.1 kOe from room temperature to various temperatures. The curves have been vertically displaced for clarity. (**b**) Exponential decay of TRM for selected temperatures. The solid line is the fit to the superposition of a stretched exponential and a constant term described in Equation (1). For each temperature, the inset shows the semi-log plot time dependence of TRM with the fit to the logarithmic relaxation described in Equation (2).

To further investigate the spin-glass behavior in the NFL/FM LCMO film, the time-dependent magnetic behavior of the sample was measured. In this case, the thermoremanent magnetization (TRM) *M(t)* was measured at several temperatures between 10 K and 300 K. For these measurements, the sample was field cooled under a magnetic field of 0.1 kOe from the paramagnetic state to the predetermined temperature. The time dependence of the field-cooled isothermal remnant magnetization [*M(t)*] was measured immediately after the cooling magnetic field was turned off. The reference time corresponded to the time at which the magnetic field was removed. It is worth mentioning that despite some uncertainty in the very early time values, the following analysis and interpretation of our experimental data were not affected. Figure 5a shows the TMR at selected temperatures that are representative of this study: T = 10 K (well below *T_B_*), T = 65 K (just below *T_B_*), T = 85 K and 100 K (below *T_P_*), T = 200 K (as representative of *T_C_* < T < *T_irr_* range), and T = 270 K (above *T_irr_*). For T = 270 K, the thermal remnant magnetization is quite small, and no clear relaxation was observed, as expected in a paramagnetic state.

Below 240 K, the TMR data indicate that the magnetization decreases with time and does not reach equilibrium on time scales up to 9000 s. Such a slow relaxation in the magnetization is detected down to the lowest recorded temperature. The TMR data were analyzed using the models describing the time-dependent relaxation of magnetization in spin glasses, e.g., the logarithmic relaxation decay and the stretched exponential decay. Those fits are shown in Figure 5b for selected temperatures. For each temperature, the solid line in the main panel displays the fit to the superposition of a stretched exponential and a constant term [32,33]:

(1) $$M\left(t\right)=M_{0}+M_{g}exp\left[-{\left(\frac{t}{\tau }\right)}^{1-n}\right]$$

where *M_0_* is related to an intrinsic ferromagnetic component and *M_g_* to a glassy component mainly contributing to the relaxation observed effects; *τ* is the characteristic relaxation time constant; and 1 − *n* is the stretching exponent, which has values between 0 and 1. In this relation, *n* = 1 implies that *M(t)* is constant, i.e., no relaxation at all, whereas *n* = 0 implies a single time-constant, exponential relaxation. The fit to the logarithmic decay is:

(2) $$M\left(t\right)=M_{0}-S\;\log\left(t\right)$$

where *M*_0_ is a constant, and *S* is the temperature-dependent magnetic viscosity [34], which is depicted in each panel as an inset.

At 200 K, the disagreement between the logarithmic fit and the observed TRM data suggests that the magnetization does not decay logarithmically at this temperature (see inset). The *M(t)* experimental data at 200 K can be better fitted to the stretched exponential behavior. The solid line in the main panel shows the best fit to Equation (1), with fitting parameters *τ* ~600 s and *n* = 0.6. These values are within the range of the different glassy systems reported [15,19,31,35,36]. It is worth mentioning that the TRM in this temperature range reflects the magnetic relaxation of the magnetic polarons in the LCMO below *T_irr_* = 240(1) K [28,29,30].

Around 100 K, a subtle change in the TRM *M(t)* is observed. Equation (1) cannot fit the data well over the full time range studied. Instead, Equation (2) gives a satisfactory fit at T < 100 K, with *M_0_* = 2.5 × 10^−4^ emu and *S* = 3.6 × 10^−7^ emu at T = 100 K (not shown). The solid line in the inset of Figure 5(b) represents the fit to the logarithmic behavior [Equation (2)], with fitting parameters *M_0_* = 2.9 × 10^−4^ emu and *S* = 2.9 × 10^−7^ emu for T = 85 K and *M_0_* = 3.6 × 10^−4^ emu and *S* = 6.2 x 10^−8^ emu for T = 10 K. Such a decay of the TRM is consistent with a spin-glass-like behavior, for which the energy barriers are randomly distributed [37,38,39,40]. It is noteworthy that this subtle change observed in the relaxation of the magnetization of the film with time occurs around the temperature below which EB has been detected in the hysteresis loops (*T_B_* ~80 K). This fact suggests that TRM in this temperature range could be reflecting a relaxation process which is different from the relaxation of the magnetic polarons in FM LCMO.

**Figure 6:**
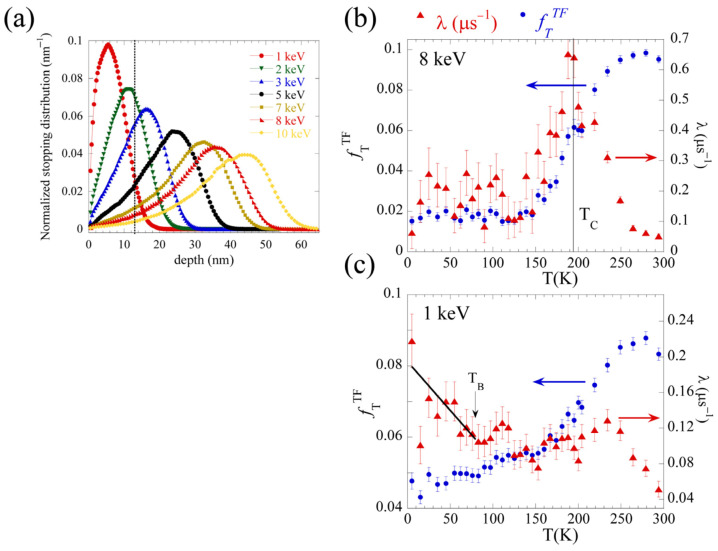
(**a**) The normalized stopping distribution of muons with different implantation energies (see inset) of a 60 nm-thick LCMO film deposited on a single-crystal STO substrate, calculated using TRIM.SP [17]. The lines are a guide for the eye. The dashed line denotes the position of the interface of the NFL with the FM layer. Temperature dependence of the muon spin relaxation rate (*λ_T_*) and the transverse fraction (*f_T_^TF^*) determined from wTF LE-μSR measurements on 60 nm-thick LCMO film for muons implanted at (**b**) FM LCMO layer (*E_imp_* = 8 keV, mean depth = 35 nm) and (**c**) NFL LCMO layer (*E_imp_* = 1 keV, mean depth = 5 nm). The solid line is a guide for the eye.

To further shed light on the magnetic nature of the NFL, we used the sensitivity and the depth-profiling capacity of low-energy muon spin spectroscopy (LE-μSR) [41,42]. On the one hand, polarized muons are very well suited for the study of spin glasses because of their high sensitivity in the time window of magnetic fluctuations of 10^−4^–10^−10^s [43]. On the other hand, by tuning the implantation energy of the muon between 1 and 30 kV, mean depths of between five nanometers and a few hundred nanometers can be chosen. Thus, we can follow the temperature dependence of the muon spin relaxation as a function of the depth below the surface of the film. Therefore, this technique allowed the study of the two spatially segregated layers (NFL-FM) with different magnetic orderings in the LCMO film and was successfully applied before in strained multiferroic 10 nm-thick SMO and SBMO thin films [1,2]. Figure 6a displays the normalized stopping distribution of muons in an LCMO film deposited on an STO substrate for different implantation energies calculated using the Monte Carlo simulation program TRIM.SP [17]. This simulation shows that the lowest muon implantation energy (*E_imp_* = 1 keV) yields a mean implantation depth of 5 nm, with 98% of the muons stopped at the 15 nm-thick layer of the LCMO films, which is optimal for the study of the NFL LCMO layer when taking into account the density of the material determined by XRR measurements. A higher implantation energy (*E_imp_* = 8 keV) was used in order to analyze the FM LCMO layer. This energy yields a mean implantation depth of about 35 nm with only 6% of the muons stopped at the NFL LCMO layer.

Temperature scans in a weak transverse magnetic field (wTF) of *B_ext_* = 10 mT applied perpendicular to the initial muon spin polarization and to the film surface were performed. It is worth mentioning that a transverse field influences some properties of the spin glass, such as the sharpness of the transition, but leaves unaltered the essential features reflecting the depth- and thickness-dependent dynamical behavior of the films [44]. In a wTF, the time evolution of the muon spin polarization is described by the relaxation function [45]:

(3) $$G_{x}\left(t\right)=f_{T}^{TF}\cos\left(\gamma_{\mu }B_{l}t+\phi \right)e^{-\lambda_{T}t}+f_{L}^{TF}e^{-\lambda_{L}t}$$

where *f_T_^TF^* and *f_L_^TF^* reflect the fraction of the muons having their spin initially transverse and longitudinal to the local magnetic field (*B_l_*) direction, respectively; *γ_μ_* is the gyromagnetic ratio of the muon; *λ_T_* and *λ_L_* are the relaxation rates, and ϕ is a phase offset. The fitting of the relaxation function *G_x_*(*t*) was performed using the *musrfit* program [46]. Above *T_C_*, *f_T_^TF^* is the full asymmetry, since only *B_ext_* is present inside the sample. Below *T_C_*, the superposition of the small external *B_ext_* and the internal magnetic fields leads to a strong dephasing of the signal; so, *f_T_^TF^* decreases to a level corresponding to the nonmagnetic fraction plus the background level. A decrease in *f_T_^TF^* demonstrates static magnetism (see Ref. [1] for details).

Figure 6b,c display the transverse fraction *f_T_^TF^* and the muon spin relaxation rate *λ_T_* as a function of temperature in the LCMO on the STO film obtained at different mean depths, i.e., with *E_imp_* = 8 keV (mean depth 35 nm) and 1 keV (mean depth 5 nm), respectively. For the muons mostly implanted in the FM layer of the LCMO film (Figure 6b), both the decrease of the transverse fraction *f_T_^TF^* (*T*) and the peak in *λ_T_*(*T*) at a characteristic temperature of ~190 K indicate the presence of a second-order transition, which, as expected, corresponds to the *T_C_* of the FM LCMO layer.

For the muons mostly implanted in the NFL layer of the LCMO film (Figure 6c), *f_T_^TF^* (*T*) shows a decrease around *T_C_*, whereas *λ_T_* increases by a factor 2–3 from the value in the paramagnetic regime, which is attributed to the presence of static stray fields originating from the FM LCMO layer.

However, unlike what is observed in the FM LCMO layer, *λ_T_* (T) increases steadily below ~100 K. A similar increase in the relaxation rate was reported in μSR studies carried out using wTF in bulk systems [47] and more recently in single layer films of AuFe and CuMn spin glasses [44] when approaching the freezing temperature *T_F_* from above, reflecting the slowing down of the magnetic moments. In the present case, such a slowing down would come from the magnetic moments in the NFL LCMO layer, which indicates the presence of a spin-glass state in this layer at temperatures lower than 100 K. It is noteworthy that the data in Figure 6c do not show any evidence of a long-range order transition in the NFL LCMO layer at temperatures down to 5 K. This observation discards the presence of an AFM state in this layer, as was previously suggested by Marin et al. to explain the origin of the EB observed at T = 10 K in these films [8]. Thus, our depth-dependent muon spin relaxation study indicates that the overall glassy behavior of the sample observed by the macroscopic magnetic measurements is due to the interaction between the FM LCMO layer with the SG region in the NFL.

Here we note that, unlike the LSMO/SMO bilayers and the LSMO/SMO/LSMO trilayers, where an SG state at the interface between the FM LSMO and the AFM SMO layers was proposed [19,20], in the present study the spin-glass behavior is ascribed to the ferromagnetically dead layer at the top of the FM LCMO layer. A similar scenario has also been proposed in Mn_5_Ge_3_ on Ge (111) single-crystalline films, where a ferromagnetically dead layer with spin-glass-like properties was reported [37] at the Mn_5_Ge_3_/Ge interface.

It is well known that frustration, which emerges as a result of site disorder, or local competition between exchange interactions, is of fundamental importance in spin glasses. As LCMO film is both chemically and structurally homogeneous [8], it is reasonable to argue that competing magnetic interactions are the source of the spin glass at the NFL on top of the FM LCMO.

The first-principle calculations of the magnetic ordering of the strained LCMO/STO reported previously [8] indicate that the FM ordering is the most stable for a tetragonality value of τ = 0, whereas a magnetic transition from an FM to an A_z_-AFM ordering is predicted for τ > 0.06. For τ = 0.025 (e.g., LCMO/STO grown at 10 Hz), however, the calculations yield a small energy difference between the FM and AFM orderings. Such a small energy difference suggests that the coexistence of the two orderings is possible in this film. Thus, the competition of the FM double-exchange interactions and the AFM interactions of similar energies would be the source of magnetic frustration in the NFL and the associated spin-glass behavior.

## 4. Conclusions

In summary, we performed a comprehensive magnetic characterization of the non-ferromagnetic layer, spatially segregated on a strained ferromagnetic LCMO film, by means of field-cooling and zero-field-cooling magnetization versus temperature measurements, field-cooled hysteresis loops, relaxation of thermoremanent magnetization, and low-energy muon spin relaxation experiments. The systematic analysis of the exchange bias points to a spin-glass behavior of the non-ferromagnetic layer in the spatially segregated epitaxial LCMO thin film around a characteristic temperature *T_B_* = 80 K. The thermoremanent magnetization measurements reflect a subtle change in the relaxation of the magnetization of the film around *T_B_*. The depth-dependent muon spin relaxation experiments evidenced the different magnetic behaviors of the two spatially segregated layers (non-ferromagnetic and ferromagnetic), in agreement with the electron holography by Marin et al. [8]. The experimental results discard the AFM nature and support the spin-glass-like scenario for the non-ferromagnetic layer. This scenario can be understood in the framework of the competing FM and AFM interactions of similar energies, as suggested by the previous DFT + U calculations of the magnetic ordering of strained LCMO.

## Data Availability

Data are available from the corresponding author upon reasonable request.
